# Notch3 Targeting: A Novel Weapon against Ovarian Cancer Stem Cells

**DOI:** 10.1155/2019/6264931

**Published:** 2019-01-06

**Authors:** Simona Ceccarelli, Francesca Megiorni, Diana Bellavia, Cinzia Marchese, Isabella Screpanti, Saula Checquolo

**Affiliations:** ^1^Department of Experimental Medicine, “Sapienza” University of Rome, Rome, Italy; ^2^Department of Pediatrics, “Sapienza” University of Rome, Italy; ^3^Department of Molecular Medicine, “Sapienza” University of Rome, Rome, Italy; ^4^Department of Medico-Surgical Sciences and Biotechnology, Sapienza University, Latina, Italy

## Abstract

Notch signaling is frequently activated in ovarian cancer (OC) and contributes to the proliferation and survival of cultured OC cells as well as to tumor formation and angiogenesis in xenograft models. Several studies demonstrate that Notch3 expression renders cancer cells more resistant to carboplatin, contributing to chemoresistance and poor survival of OC-bearing patients. This suggests that Notch3 can represent both a biomarker and a target for therapeutic interventions in OC patients. Although it is still unclear how chemoresistance arises, different lines of evidence support a critical role of cancer stem cells (CSCs), suggesting that CSC targeting by innovative therapeutic approaches might represent a promising tool to efficiently reduce OC recurrence. To date, CSC-directed therapies in OC tumors are mainly targeted to the inhibition of CSC-related signaling pathways, including Notch. As it is increasingly evident the involvement of Notch signaling, and in particular of Notch3, in regulating stem-like cell maintenance and expansion in several tumors, here we provide an overview of the current knowledge of Notch3 role in CSC-mediated OC chemoresistance, finally exploring the potential design of innovative Notch3 inhibition-based therapies for OC treatment, aimed at eradicating tumor through the suppression of CSCs.

## 1. Introduction

Ovarian cancer (OC) is relatively rare (nearly 3% of all female tumors) but it represents the most lethal gynecologic malignancy worldwide, being the fifth principal cause of cancer mortality in women [1]. About 200,000 new OC cases are estimated worldwide every year, with 150,000 deaths [2]. This high mortality-to-incidence ratio is essentially due to the absence of OC-specific symptoms and the lack of effective screening strategies that lead many women to be diagnosed at an advanced stage of the disease, when cancer metastases are already present in the abdominal cavity [3]. Currently, despite the standard therapies, including cytoreduction and platinum/paclitaxel administration, which may lead to clinical remission, about 70% of patients relapse developing a resistance to first-line drugs [4]. Indeed, the general prognosis in OC patients remains poor, with a 5-year survival rate of about 50% [1].

In this scenario, the high percentage of therapeutic failure is mainly due to the occurrence of drug resistance, which is directly correlated with the presence of cancer stem cells (CSCs) [5–7]. Molecular profiling of ovarian CSCs has been performed to identify both stemness-related surface markers and molecular pathways important for CSC function [6]. Besides the high number of genes related to drug efflux and DNA damage repair, which contribute to CSC resistance to conventional chemotherapy, the specific role of Notch signaling pathway, mainly of Notch3, has been well established in the regulation of CSC behavior and platinum chemoresistance [6]. Notch signaling is a conserved pathway commonly implicated in the maintenance of tissue homeostasis by regulating self-renewal of stem cells and differentiation of progenitor cells in several organs and tissues [8–10]. More recently, it has been demonstrated a novel specific role of Notch3 in neural stem cell maintenance in the adult brain, through a dual control of both stemness and quiescence [11].

In keeping with this, Notch signaling dysregulation is correlated with the acquisition and maintenance of CSC-like properties in several tumors [12, 13], including brain [14], lung [15], breast [16], liver [17] and ovarian cancers [6], thus suggesting its emerging role as an attractive therapeutic target, as Notch inhibition may allow the elimination of CSCs.

Notch3, one of the four Notch receptors triggering the Notch signaling pathway, has been reported as a candidate oncogene overexpressed in more than 20% of ovarian serous adenocarcinomas. In different tumor contexts, including ovarian cancer, its deregulated expression was found to be correlated with tumor recurrence, drug resistance, and a poor prognostic outcome [7, 18–21].

In this review, we draw a picture on the current knowledge about the role of Notch3 in ovarian CSC behavior regulation, in order to suggest potential Notch3 inhibition-based cancer treatments aimed at improving the prognosis of OC patients.

## 2. CSCs in Ovarian Cancer

In recent years, several models of CSC biology have been proposed to explain how tumor heterogeneity develops and contributes to the early stage of tumor formation, disease progression, and drug resistance [22]. The CSC intratumoral heterogeneity is a common feature described in several tumors and OC represents one of the best examples [23, 24]. Indeed, tumor masses are composed of different cell types, recognized upon their phenotype and function, and CSCs represent a small subset of tumor cells, possessing self-renewing properties. Furthermore, CSCs are also able to generate nontumorigenic cell progeny, also called non-CSC [25], which makes up most of the tumor cell population and encompasses distinct genetic and epigenetic characteristics [26]. More recently, it has been also suggested a model by which CSCs could acquire the ability of self-renewal through the dedifferentiation of progenitor cells and the phenotype reversal of terminally differentiated cells [27]. Moreover, it has been proposed that tumor microenvironment can trigger specific signals that are able to change the phenotype of both CSCs and differentiated cells independently from genetic mechanisms (i.e., mutations), thus making them interconvertible [28–30].

Altogether, these data support the general notion that the acquisition of genetic and epigenetic alterations by CSCs and the influence of the specific microenvironment determine the ability of CSCs to evade the systemic effects of standard chemotherapies, thus mediating drug resistance and cancer recurrence [31–34]. Although CSC targeting would be an interesting option to overcome drug resistance, it is still a big challenge. Understanding and further dissecting the molecular mechanisms that control CSCs as well as the specific markers for appropriate CSC identification, isolation and characterization can have important implications for improving cancer therapies. In this regard, also other important aspects in CSC biology, such as the tumor cell origin [35] and the role of microenvironment [36], need to be further studied and considered.

Mouse model-derived established OC cell lines and patient-derived samples of OC were used for ovarian CSC isolation by using a number of cell surface markers, including CD133, CD44, CD24, CD117, ALDH1A1, and EpCAM [34, 37]. However, it has been reported that several of these are not uniformly useful in identifying CSCs because they are not exclusively expressed by OC tissues [38]. An exhaustive review of ovarian CSC marker expression and function has been recently published [39]. The heterogeneity of the identified ovarian CSC putative markers could be due to different factors, mainly related to the heterogeneity of the disease itself, to the existence of different pools of ovarian CSCs and to their high genetic and phenotypic plasticity [39]. Therefore, the use of combinatorial markers and the need for more specific markers and revelation techniques to detect ovarian CSCs are urgent.

In this regard, an increasing number of studies have demonstrated the important role of the side population (SP) cells in the identification of ovarian CSCs, as SP cells are able to maintain a typical “CSC phenotype,” including regenerative and self-renewing capacities, tumorigenicity, and resistance to therapy [34, 40–43]. However, it has been also demonstrated that SP cells derived from different OC cell lines could express different markers, thus defining a potentially heterogeneous CSC compartment [23]. Therefore, further investigations are required to isolate SP cells possessing CSC properties with respect to non-SP cells, in order to evolve the knowledge of CSC-based therapies for future OC treatment.

Several lines of evidence have demonstrated that CSCs may confer growth advantage and metastatic properties to chemoresistant ovarian tumors [34, 44, 45] through complex mechanisms, which are not fully understood as yet. CSCs may be related with several mechanisms including cell cycle arrest, increased DNA protection, repair enzyme system, and inherent epigenetic aberrations [46]. Furthermore, it has been demonstrated that the decreased chemotherapy responsiveness of CSCs could be also due to the activation of several CSC-related prosurvival signaling pathways, such as Wnt/*β*-catenin, IL6/JAK/STAT3, Hedgehog, NF*κ*B, PI3K/AKT, PDGFR, and Notch [46, 47], making them possible key targets for eradicating CSC populations and impairing metastatic behavior.

The OC therapy could be strongly supported by research derived from CSC characterization, aimed at identifying compounds that show significant potential for future personalized medicine based on the development of ovarian CSC-specific therapeutic agents [48, 49].

## 3. Notch3 Signaling Overview

Notch signaling pathway includes receptors, ligands, positive and negative modifiers as well as various transcription factors. In the vertebrates, four Notch receptors (Notch 1-4) and two ligand families (delta-like 1, 3, 4 and Jagged 1, 2) were identified. From a structural point of view, Notch receptors are transmembrane proteins, consisting of an extracellular portion which is rich in the epidermal growth factor (EGF) repeats, a transmembrane domain and an intracellular domain [49]. The canonical Notch signaling is triggered when a Notch receptor interacts with a ligand expressed by a neighboring cell. This binding leads to Notch cleavage by a protease of the ADAM family, thus releasing its extracellular portion and generating the substrate for subsequent cleavage of the Notch receptor intracellular domain (NICD) by the *γ*-secretase complex. After this, the NICD translocates to the nucleus where it interacts with a transcription factor, called CSL (for CBF-1/RBP-J*κ*, Suppressor of Hairless and Lag-1), and a transcriptional coactivator of the mastermind-like (MAML) family to form a transcriptional activation complex [49].

### 3.1. Notch3 and Ovarian Cancer

The Notch signaling pathway has been implicated in numerous human malignancies [50]. In particular, Notch3 pathway dysregulation is often associated with the pathogenesis and progression of several tumors [51–54], including OC [55]. The amplification of the chr19p13.12 region, encompassing the Notch3 locus, and the upregulation of Notch3 expression, both at mRNA and protein levels, have been detected in a large percentage of OC [56]. Notch3-overexpressing tumors require Notch3 for proliferation and survival, thus suggesting that the specific inactivation of Notch3 can represent a potential therapeutic approach for OC patients. Indeed, the selective downregulation of Notch3 by using either Notch3-specific small interfering RNA (siRNA) or *γ*-secretase inhibitors (GSIs) significantly reduced cell proliferation and induced apoptosis in Notch3-overexpressing cell lines but not in cell lines that expressed a minimal amount of Notch3 [56]. Park and colleagues further demonstrated that Notch3 activation is strongly correlated with carboplatin resistance, as retrovirally driven NICD3 transduced in OC cells was sufficient to increase cell survival upon carboplatin treatment [7]. Interestingly, the presence of nuclear NICD3 was observed more frequently in postchemotherapy recurrent ovarian serous carcinomas than in their primary counterparts, thus suggesting that the activation of Notch3 signaling may be advantageous for cancer cell survival under the selection pressure of chemotherapy [7]. In addition, NICD3 overexpression in OC cells resulted in the upregulation of several stem cell markers, such as Nanog, Oct4, Klf4, Rex1, and NAC1, as well as of ABCB1 gene, which is a member of the superfamily of ATP-binding cassette (ABC) transporters, known to be involved in multidrug resistance through a mechanism of drug efflux pumping [57]. In agreement with these data, the CSC markers ALDH1A1 and CD44, which were found highly expressed in non-small-cell lung cancer (NSCLC) patients with chemoresistance, were positively correlated with Notch3 expression [58]. Notably, Notch3 blockade led to the prevention of the autophagy process commonly related to chemoresistance of CSCs [58].

Furthermore, it has been also demonstrated that Notch3 inhibition abrogated the colony and tumor-forming ability of ALDH-positive cells in lung cancer [59, 60]. Consistent with these data, Ali and colleagues provided significant mechanistic insights into how Notch3 was able to drive a stem-like phenotype and tumorigenesis ability of KRAS lung adenocarcinoma (LDAC), the most prevalent form of lung cancer characterized by poor therapeutic response and high relapse rate [28].

### 3.2. Notch3 and Ovarian Cancer Stem Cells

It has been recently demonstrated that Notch3 is overexpressed in ALDH1^+^ ovarian cells [61], thus supporting again the link between Notch3 and CSC function, as ADLH1 is reported to be the most potent ovarian CSC marker [62] and high expression of ALDH1 mRNA was associated with advanced clinical stage and chemoresistance [63]. Interestingly, Kim and colleagues showed for the first time the clinical significance of Notch3 and ADHL1 coexpression observed in human OC tissues, suggesting that their combined upregulation represents an independent poor prognostic indicator in OC [61].

The acquisition of a “stemness phenotype” through a Notch3-dependent mechanism suggests a link between Notch3 activation and drug resistance, and therefore tumor recurrence, as stem cell-like properties are known to be involved in chemoresistance development.

In keeping with these data, Kang and colleagues showed that Notch inhibition in paclitaxel-resistant ovarian cancer cells significantly results in reduced viability, migration, and angiogenesis and increased apoptosis. Notably, both pan-Notch inhibitors GSIs and Notch3 siRNA treatments induce decreased spheroid formation, which represents the self-renewal ability of CSCs, accompanied by a significant downregulation of stem cell markers such as ALDH1, CD24, CD44, CD133, SOX2, and c-kit (CD117) in resistant cells, thus indicating that Notch3 blocking inhibits ovarian CSC activation. Together, these data demonstrated that Notch3-specific inhibition resensitizes paclitaxel-resistant ovarian cancer cells to paclitaxel with an efficacy comparable to GSIs [64].

The studies by McAuliffe and colleagues strongly supported the specific role of Notch3 signaling pathway in CSC maintenance and tumor resistance to platinum both *in vitro* and *in vivo* [6]. By using animal models and human tumor samples, the authors fully characterized the side population, previously isolated for their ability to efflux the Hoechst 33342 fluorescent dye [65], demonstrating that SP cells are enriched for ovarian CSCs, based on their transcriptional profile, which showed upregulation of gene coding for key stem cell surface markers, multidrug transporters, self-renewal, DNA repair, angiogenesis, and differentiation. Furthermore, CSCs overexpress high levels of genes associated with pivotal signaling pathways, including Notch, TGF-*β*, IGF, EGF, FGF, and WNT/*β*-catenin [6]. OC SP cells are more tumorigenic than non-SP (NSP) cells *in vivo* and display an increased resistance to platinum [6]. Interestingly, the authors demonstrated that standard platinum therapy targets mostly NSP cells, whereas GSIs target Notch-dependent cells, including SP. They showed that only the cisplatin/GSI combined treatments may be effective in targeting both CSCs and the bulk of tumor cells, thus being critical for tumor eradication, and finally increasing platinum response and cell death by enhancing the DNA damage response. Notably, the sensitivity to GSIs was correlated with the presence of Notch3 gene expression, thus further confirming the same results obtained by using Notch3 siRNA knockdown [6]. Interestingly, they also suggested including Notch inhibitors in the first-line platinum treatment of OC to achieve the best therapeutic results, as they observed that relapsed tumor cells, including CSCs, were no longer completely dependent on the Notch signaling pathway [6]. In keeping with this, the usefulness of Notch3 as a prognostic biomarker and a therapeutic target in patients with advanced stage OC has been suggested, since the overexpression of NICD3 observed in primary OC was also highly predictive of a shorter progression-free interval [66].

**Figure 1:**
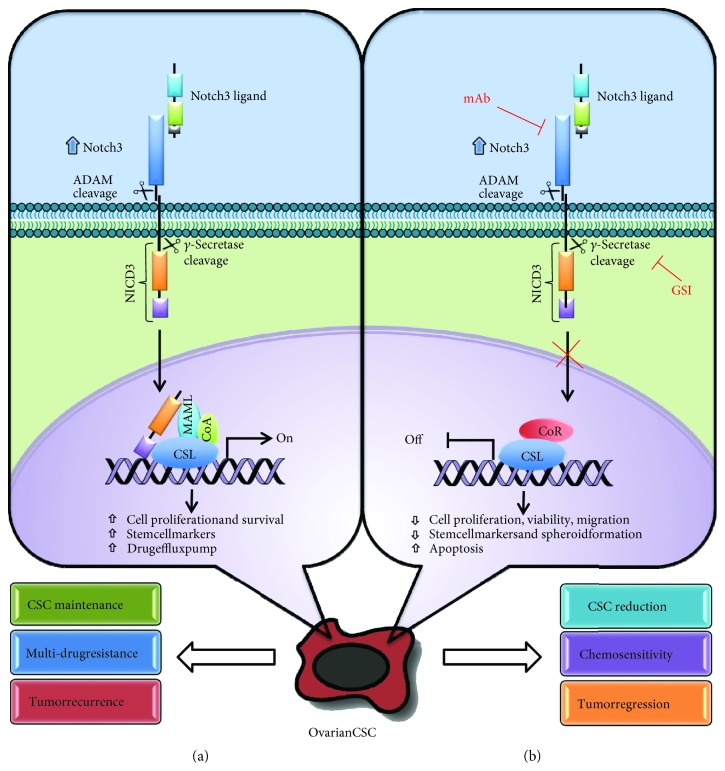
Diagram of the Notch3 signaling pathway in ovarian CSC. (a) In Notch3-overexpressing ovarian CSC, Notch signaling is triggered by ligand binding to Notch3 receptor, which undergoes a two-step proteolytic cleavage by ADAM family proteases and *γ*-secretases, respectively. Subsequently, the Notch3 intracellular domain (NICD3) is released and translocates into the nucleus, where it binds to CSL and converts the transcriptional complex from a repressor to an activator of Notch3 target genes, known to be implicated in CSC maintenance, drug resistance and tumor recurrence. (b) Notch3 signaling inhibition by *γ*-secretase inhibitors (GSIs) or monoclonal antibodies against Notch3 blocks Notch3 target gene activation, resulting in the reduction of CSCs, increased chemosensitivity and tumor regression.

All these studies support the notion that Notch3 activation may reprogram tumor cells to assume a stem-like profile and contribute to platinum chemoresistance in OC, which is responsible for tumor recurrence. These findings may have important therapeutic implications in the treatment of OC patients (Figure 1).

## 4. Conclusion

One of the critical challenges in the treatment of patients with OC is the development of chemoresistant recurrent disease, mainly due to the presence of a higher proportion of CSCs with drug-resistance phenotype embedded within bulk tumors. For this reason, there is considerable interest in the development of new compounds that may be therapeutically effective against this insidious subpopulation of tumor cells.

Several studies correlated Notch3 with CSC activation and maintenance, thus suggesting that NICD3 expression may represent a novel clinical parameter for the prediction of patient survival and providing a rationale for future development of Notch3-based therapy for OC.

Besides its central role in T-cell acute leukemia (T-ALL) development, the activation of Notch signaling actually represents a possible target in several solid tumors, and for this reason a number of novel Notch inhibitory strategies have been undertaken [67].

Targeting the Notch pathway either with small molecules, acting as *γ*-secretase inhibitors (GSIs), or large molecules, such as monoclonal antibodies (mAbs) against Notch receptors, is in clinical development [68]. Although GSI treatment has progressed into the clinic, it fails to distinguish individual Notch receptors and causes intestinal toxicity, attributed to the dual inhibition of Notch1 and Notch2 [69]. Therefore, the specific inhibition of a single Notch receptor or ligand might provide a less toxic alternative for Notch inhibition.

Multiple studies have demonstrated that specific Notch3 inhibition sensitizes tumor cells to chemotherapy in drug-resistant OC, with an efficacy comparable to GSIs [6, 64]. Furthermore, it has been recently demonstrated that miR-136 overexpression, by directly targeting Notch3, resensitized paclitaxel-resistant OC cells and reduced CSC activities [70]. Actually, miRNA-targeting therapies are in preclinical and clinical development for the treatment of different diseases and cancers and targeting miRNAs related to Notch would be a valuable therapeutic option in OC treatment.

**Figure 2:**
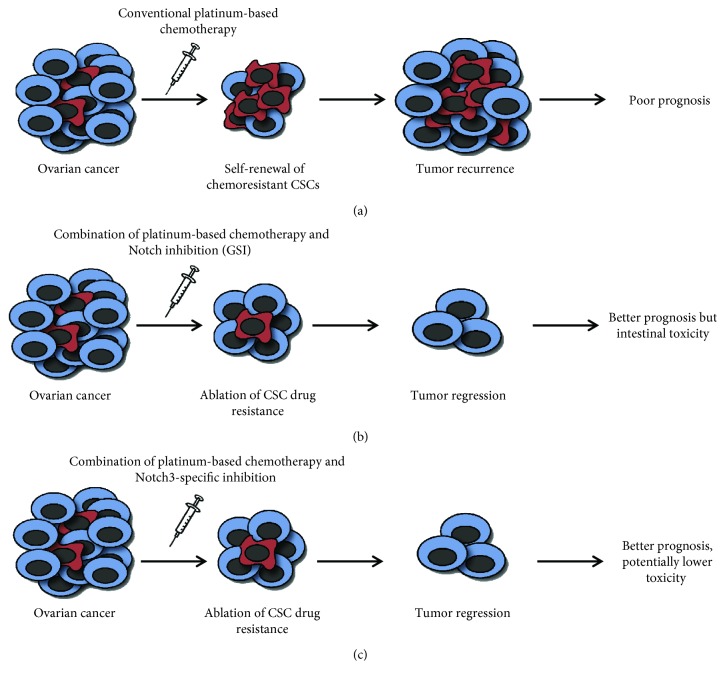
A proposed model for therapeutic approaches targeting ovarian CSC. (a) Conventional chemotherapy targets the cells that constitute the bulk of the tumor, but CSCs frequently develop drug resistance and their subsequent enrichment generally leads to tumor recurrence and poor prognosis. (b) Combined therapies involving inhibition of Notch signaling by using conventional drugs, such as GSI as pan-Notch inhibitor, are able to sensitize CSCs to chemotherapy and ameliorate patients' prognosis but are associated with intestinal toxicity. (c) Combined therapies, targeting CSCs through the specific Notch3 inhibition, can potentially result in tumor regression and reduced toxicity.

Together, all these findings strongly support the importance of new clinical trials aimed at evaluating more selective and less toxic Notch3-based specific therapies in increasing sensitivity to platinum treatment, finally improving the outcomes of OC patients (Figure 2).

## References

[1] Siegel R. L., Miller K. D., Jemal A. (2016). Cancer statistics, 2016. *CA: a Cancer Journal for Clinicians*.

[2] Reid B. M., Permuth J. B., Sellers T. A. (2017). Epidemiology of ovarian cancer: a review. *Cancer Biology & Medicine*.

[3] Matulonis U. A., Sood A. K., Fallowfield L., Howitt B. E., Sehouli J., Karlan B. Y. (2016). Ovarian cancer. *Nature Reviews Disease Primers*.

[4] Cannistra S. A. (2004). Cancer of the ovary. *The New England Journal of Medicine*.

[5] Gupta P. B., Onder T. T., Jiang G. (2009). Identification of selective inhibitors of cancer stem cells by high-throughput screening. *Cell*.

[6] McAuliffe S. M., Morgan S. L., Wyant G. A. (2012). Targeting Notch, a key pathway for ovarian cancer stem cells, sensitizes tumors to platinum therapy. *Proceedings of the National Academy of Sciences of the United States of America*.

[7] Park J. T., Chen X., Tropè C. G., Davidson B., Shih I. M., Wang T. L. (2010). Notch3 overexpression is related to the recurrence of ovarian cancer and confers resistance to carboplatin. *The American Journal of Pathology*.

[8] Chiba S. (2006). Concise review: Notch signaling in stem cell systems. *Stem Cells*.

[9] Rieskamp J. D., Denninger J. K., Dause T. J. (2018). Identifying the unique role of Notch3 in adult neural stem cell maintenance. *The Journal of Neuroscience*.

[10] Wang Z., Li Y., Banerjee S., Sarkar F. H. (2009). Emerging role of Notch in stem cells and cancer. *Cancer Letters*.

[11] Than-Trong E., Ortica-Gatti S., Mella S., Nepal C., Alunni A., Bally-Cuif L. (2018). Neural stem cell quiescence and stemness are molecularly distinct outputs of the Notch3 signalling cascade in the vertebrate adult brain. *Development*.

[12] Ranganathan P., Weaver K. L., Capobianco A. J. (2011). Notch signalling in solid tumours: a little bit of everything but not all the time. *Nature Reviews Cancer*.

[13] Tang C., Ang B. T., Pervaiz S. (2007). Cancer stem cell: target for anti-cancer therapy. *The FASEB Journal*.

[14] Fan X., Khaki L., Zhu T. S. (2010). NOTCH pathway blockade depletes CD133-positive glioblastoma cells and inhibits growth of tumor neurospheres and xenografts. *Stem Cells*.

[15] Ali S. A., Justilien V., Jamieson L., Murray N. R., Fields A. P. (2016). Protein kinase Cι drives a NOTCH3-dependent stem-like phenotype in mutant *KRAS* lung adenocarcinoma. *Cancer Cell*.

[16] Rustighi A., Zannini A., Tiberi L. (2014). Prolyl-isomerase Pin1 controls normal and cancer stem cells of the breast. *EMBO Molecular Medicine*.

[17] Liu C., Liu L., Chen X. (2018). LSD1 stimulates cancer-associated fibroblasts to drive Notch3-dependent self-renewal of liver cancer stem-like cells. *Cancer Research*.

[18] Arasada R. R., Amann J. M., Rahman M. A., Huppert S. S., Carbone D. P. (2014). EGFR blockade enriches for lung cancer stem-like cells through Notch3-dependent signaling. *Cancer Research*.

[19] Diluvio G., del Gaudio F., Giuli M. V. (2018). NOTCH3 inactivation increases triple negative breast cancer sensitivity to gefitinib by promoting EGFR tyrosine dephosphorylation and its intracellular arrest. *Oncogene*.

[20] Hu S., Fu W., Li T. (2017). Antagonism of EGFR and Notch limits resistance to EGFR inhibitors and radiation by decreasing tumor-initiating cell frequency. *Science Translational Medicine*.

[21] Jung S. G., Kwon Y. D., Song J. A. (2010). Prognostic significance of Notch 3 gene expression in ovarian serous carcinoma. *Cancer Science*.

[22] Del Re M., Arrigoni E., Restante G. (2018). Concise review: resistance to tyrosine kinase inhibitors in non‐small cell lung cancer: the role of cancer stem cells. *Stem Cells*.

[23] Boesch M., Zeimet A. G., Reimer D. (2014). The side population of ovarian cancer cells defines a heterogeneous compartment exhibiting stem cell characteristics. *Oncotarget*.

[24] Tomao F., Papa A., Strudel M. (2014). Investigating molecular profiles of ovarian cancer: an update on cancer stem cells. *Journal of Cancer*.

[25] Dick J. E. (2009). Looking ahead in cancer stem cell research. *Nature Biotechnology*.

[26] Li Y., Laterra J. (2012). Cancer stem cells: distinct entities or dynamically regulated phenotypes?. *Cancer Research*.

[27] Friedmann-Morvinski D., Verma I. M. (2014). Dedifferentiation and reprogramming: origins of cancer stem cells. *EMBO Reports*.

[28] Boesch M., Sopper S., Zeimet A. G. (2016). Heterogeneity of cancer stem cells: rationale for targeting the stem cell niche. *Biochimica et Biophysica Acta (BBA) - Reviews on Cancer*.

[29] Melzer C., von der Ohe J., Lehnert H., Ungefroren H., Hass R. (2017). Cancer stem cell niche models and contribution by mesenchymal stroma/stem cells. *Molecular Cancer*.

[30] Plaks V., Kong N., Werb Z. (2015). The cancer stem cell niche: how essential is the niche in regulating stemness of tumor cells?. *Cell Stem Cell*.

[31] Liu L., Li W.-Y., Chen Q., Zheng J.-K., Yang L.-Y. (2012). The biological characteristics of glioma stem cells in human glioma cell line SHG44. *Molecular Medicine Reports*.

[32] Polyak K., Hahn W. C. (2006). Roots and stems: stem cells in cancer. *Nature Medicine*.

[33] Varas-Godoy M., Rice G., Illanes S. E. (2017). The crosstalk between ovarian cancer stem cell niche and the tumor microenvironment. *Stem Cells International*.

[34] Zhang S., Balch C., Chan M. W. (2008). Identification and characterization of ovarian cancer-initiating cells from primary human tumors. *Cancer Research*.

[35] Visvader J. E. (2011). Cells of origin in cancer. *Nature*.

[36] Borovski T., de Sousa E Melo F., Vermeulen L., Medema J. P. (2011). Cancer stem cell niche: the place to be. *Cancer Research*.

[37] Baba T., Convery P. A., Matsumura N. (2009). Epigenetic regulation of CD133 and tumorigenicity of CD133+ ovarian cancer cells. *Oncogene*.

[38] Deng J., Wang L., Chen H. (2016). Targeting epithelial-mesenchymal transition and cancer stem cells for chemoresistant ovarian cancer. *Oncotarget*.

[39] Lupia M., Cavallaro U. (2017). Ovarian cancer stem cells: still an elusive entity?. *Molecular Cancer*.

[40] Gao M. Q., Choi Y. P., Kang S., Youn J. H., Cho N. H. (2010). CD24+ cells from hierarchically organized ovarian cancer are enriched in cancer stem cells. *Oncogene*.

[41] Hu L., McArthur C., Jaffe R. B. (2010). Ovarian cancer stem-like side-population cells are tumourigenic and chemoresistant. *British Journal of Cancer*.

[42] Kobayashi Y., Seino K. I., Hosonuma S. (2011). Side population is increased in paclitaxel-resistant ovarian cancer cell lines regardless of resistance to cisplatin. *Gynecologic Oncology*.

[43] Yasuda K., Torigoe T., Morita R. (2013). Ovarian cancer stem cells are enriched in side population and aldehyde dehydrogenase bright overlapping population. *PLoS One*.

[44] Bapat S. A., Mali A. M., Koppikar C. B., Kurrey N. K. (2005). Stem and progenitor-like cells contribute to the aggressive behavior of human epithelial ovarian cancer. *Cancer Research*.

[45] Steg A. D., Bevis K. S., Katre A. A. (2012). Stem cell pathways contribute to clinical chemoresistance in ovarian cancer. *Clinical Cancer Research*.

[46] Pattabiraman D. R., Weinberg R. A. (2014). Tackling the cancer stem cells — what challenges do they pose?. *Nature Reviews. Drug Discovery*.

[47] Matsui W. H. (2016). Cancer stem cell signaling pathways. *Medicine*.

[48] Mezencev R., Wang L., McDonald J. F. (2012). Identification of inhibitors of ovarian cancer stem-like cells by high-throughput screening. *Journal of Ovarian Research*.

[49] Palermo R., Checquolo S., Bellavia D., Talora C., Screpanti I. (2014). The molecular basis of notch signaling regulation: a complex simplicity. *Current Molecular Medicine*.

[50] Espinoza I., Miele L. (2013). Notch inhibitors for cancer treatment. *Pharmacology & Therapeutics*.

[51] Franciosa G., Diluvio G., Gaudio F. D. (2016). Prolyl-isomerase Pin1 controls Notch3 protein expression and regulates T-ALL progression. *Oncogene*.

[52] Checquolo S., Palermo R., Cialfi S. (2010). Differential subcellular localization regulates c-Cbl E3 ligase activity upon Notch3 protein in T-cell leukemia. *Oncogene*.

[53] Bellavia D., Mecarozzi M., Campese A. F., Grazioli P., Gulino A., Screpanti I. (2007). Notch and Ikaros: not only converging players in T cell leukemia. *Cell Cycle*.

[54] Campese A. F., Grazioli P., Colantoni S. (2009). Notch3 and pT*α*/pre-TCR sustain the *in vivo* function of naturally occurring regulatory T cells. *International Immunology*.

[55] The Cancer Genome Atlas Research Network (2011). Integrated genomic analyses of ovarian carcinoma. *Nature*.

[56] Park J. T., Li M., Nakayama K. (2006). *Notch3* gene amplification in ovarian cancer. *Cancer Research*.

[57] Higgins C. F. (2007). Multiple molecular mechanisms for multidrug resistance transporters. *Nature*.

[58] Ma Y., Li M., Si J. (2016). Blockade of Notch3 inhibits the stem-like property and is associated with ALDH1A1 and CD44 via autophagy in non-small lung cancer. *International Journal of Oncology*.

[59] Hassan K. A., Wang L., Korkaya H. (2013). Notch pathway activity identifies cells with cancer stem cell-like properties and correlates with worse survival in lung adenocarcinoma. *Clinical Cancer Research*.

[60] Sullivan J. P., Spinola M., Dodge M. (2010). Aldehyde dehydrogenase activity selects for lung adenocarcinoma stem cells dependent on notch signaling. *Cancer Research*.

[61] Kim M. J., Kim A. R., Jeong J. Y. (2017). Correlation of *ALDH1* and *Notch3* expression: clinical implication in ovarian carcinomas. *Journal of Cancer*.

[62] Silva I. A., Bai S., McLean K. (2011). Aldehyde dehydrogenase in combination with CD133 defines angiogenic ovarian cancer stem cells that portend poor patient survival. *Cancer Research*.

[63] Park Y. T., Jeong J. Y., Lee M. J. (2013). MicroRNAs overexpressed in ovarian ALDH1-positive cells are associated with chemoresistance. *Journal of Ovarian Research*.

[64] Kang H., Jeong J. Y., Song J. Y. (2016). Notch3-specific inhibition using siRNA knockdown or GSI sensitizes paclitaxel-resistant ovarian cancer cells. *Molecular Carcinogenesis*.

[65] Szotek P. P., Pieretti-Vanmarcke R., Masiakos P. T. (2006). Ovarian cancer side population defines cells with stem cell-like characteristics and Mullerian inhibiting substance responsiveness. *Proceedings of the National Academy of Sciences of the United States of America*.

[66] Rahman M. T., Nakayama K., Rahman M. (2012). Notch3 overexpression as potential therapeutic target in advanced stage chemoresistant ovarian cancer. *American Journal of Clinical Pathology*.

[67] Bellavia D., Palermo R., Felli M. P., Screpanti I., Checquolo S. (2018). Notch signaling as a therapeutic target for acute lymphoblastic leukemia. *Expert Opinion on Therapeutic Targets*.

[68] Takebe N., Nguyen D., Yang S. X. (2014). Targeting notch signaling pathway in cancer: clinical development advances and challenges. *Pharmacology & Therapeutics*.

[69] van Es J. H., van Gijn M. E., Riccio O. (2005). Notch/*γ*-secretase inhibition turns proliferative cells in intestinal crypts and adenomas into goblet cells. *Nature*.

[70] Jeong J. Y., Kang H., Kim T. H. (2017). MicroRNA-136 inhibits cancer stem cell activity and enhances the anti-tumor effect of paclitaxel against chemoresistant ovarian cancer cells by targeting Notch3. *Cancer Letters*.

